# Lysozyme as an alternative to antibiotics improves growth, antioxidants status, immunity, and intestinal bacteria in broiler chickens during the fattening period

**DOI:** 10.5194/aab-67-185-2024

**Published:** 2024-04-18

**Authors:** Ibrahim T. El-Ratel, Mahmoud H. EL-Deep, Nada K. Alharbi, Worood A. A. Alyoubi, Khaled H. El-Kholy, Ahmed A. Badawy, Ahmed Ezzat Ahmed, Mohammed F. M. El Basuini, Mahmoud Alagawany, Sara F. Fouda

**Affiliations:** 1 Department of Animal, Poultry and Fish Production, Faculty of Agriculture, Damietta University, Damietta 7952567, Egypt; 2 Animal Production Research Institute, Agricultural, Research Center, Ministry of Agriculture, Giza 12513, Egypt; 3 Department of Biology, College of Science, Princess Nourah bint Abdulrahman University, P.O. Box 84428, Riyadh 11671, Saudi Arabia; 4 Biological Sciences Department, College of Science and Arts, King Abdulaziz University, Rabigh 21911, Saudi Arabia; 5 Department of Biology, College of Science, King Khalid University, Abha 61413, Saudi Arabia; 6 Prince Sultan Bin Abdelaziz for Environmental Research and Natural Resources Sustainability Center, King Khalid University, Abha 61421, Saudi Arabia; 7 Department of Animal Production, Faculty of Agriculture, Tanta University, Tanta 31527, Egypt; 8 Faculty of Desert Agriculture, King Salman International University, South Sinai 46511, Egypt; 9 Poultry Department, Agriculture Faculty, Zagazig University, Zagazig 44511, Egypt; 10 Department of Poultry Production, Faculty of Agriculture, Mansoura University, Mansoura 35516, Egypt

## Abstract

The present study aimed to evaluate the impact of dietary lysozyme levels on the growth performance, hematological and blood biochemical parameters, immunity, antioxidant capacity, and intestinal microbial count in broiler chickens. Three-hundred 1 d old birds (Cobb-avian500) were used and divided into five groups (five replicates per group, 60 birds per replicate). Birds in the first group were fed a control diet, while birds in the second, third, fourth, and fifth groups were fed the control diet with 0.2 g lincomycin, 1 g commercial lysozyme, 25 mg chicken egg lysozyme, and 50 mg egg lysozyme per kg of diet, respectively. Results confirmed that, in comparison with the control diet, all supplements had greater impacts on final body weight and body weight gain, and only the egg lysozyme diet (50 mg kg ${}^{-1}$ diet) increased feed intake. Lincomycin, commercial lysozyme, and egg lysozyme (25 mg) improved the feed conversion ratio (FCR). Birds fed commercial lysozyme and egg lysozymes showed significantly increased hemoglobin and red blood cell counts. All supplements reduced white blood cells, heterophils, and heterophils / lymphocytes ratio and increased lymphocytes. All supplements significantly increased serum total protein, albumin, globulin, and glucose. The diet of egg lysozyme (50 mg kg ${}^{-1}$ ) significantly decreased alpha-globulin, alanine aminotransferase, triglycerides, cholesterol, and urea levels and increased high-density lipoproteins. Diets with lincomycin, commercial lysozyme, and egg lysozyme significantly increased antioxidant capacity and decreased malondialdehyde (MDA). The interferon-gamma (IFN γ ) and interlukin-2 (IL-2) were significantly improved by lincomycin, commercial lysozyme and egg lysozyme diets, but interlukin-10 (IL-10) was significantly increased only by the egg lysozyme (50 mg kg ${}^{-1}$ ) diet. The total bacterial count, *Salmonella*, *Escherichia coli*, and *Proteus* counts were significantly decreased. Dressing rate and breast weight percentage were significantly increased by the egg lysozyme (50 mg kg ${}^{-1}$ ) diet. Thigh weight percentage was increased only by the commercial lysozyme diet. In conclusion, chicken egg lysozyme (50 mg kg ${}^{-1}$ ), a promising alternative for antibiotics in broiler chickens' diet, can enhance growth performance, antioxidant status, immunity, and intestinal bacteria.

## 1 Introduction

In agriculture and veterinary medicine, poultry production is one of the most prominent fields due to the importance of increasing the requirements for meat and eggs with relatively low cost (Dhama et al., 2015; Alagawany et al., 2020). The poultry industry has grown along with the usual usage of antibiotics to high profits and making the cost of production more effective (Rafiq et al., 2022; Abd El-Hack and Alagawany, 2022). Gut bacteria significantly affect the host's health and induce many diseases (Turnbaugh et al., 2006). Gut microbial communities enhance chicken health via vitamin synthesis, feed digestion, and boosting the immune system (Abdel-Latif et al., 2017). Anti-nutritional agents deleteriously affect bacteria and immune response, and alterations in microbiota may decrease these impaired effects, enhance intestinal morphology, and reduce the free radicals in the gut (Lallès, 2016).

Antibiotics are the most effective agent causing disorders in the intestinal microbial communities in humans and animals (Lozupone et al., 2012). In the poultry industry, growth promoters with antibiotics have been used for several years to develop healthy gut, growth efficiency, and health status of chickens (Zhang et al., 2021; Rafiq et al., 2022). Many safe and effective alternative dietary additives have been estimated for their ability to replace antibiotics as growth promoters in diets without the stigma associated with their use (Rafiq et al., 2022). In this way, several reports have been carried out to look for pro- and prebiotics, herbs, and exogenous enzymes as natural agents instead of antibiotics that mimic the similar positive effects of growth parameters, antioxidant capacity, and the immune system (Patterson and Burkholder, 2003; Guo et al., 2004; Abdel-Latif et al., 2017).

Lysozyme (1,4- β -N-acetylmuramidase) enzymatically cleaves a glycosidic linkage in the peptidoglycan components of the bacterial cell wall, leading to the loss of cell membrane integrity and cellular death (Ellison and Giehl, 1991; Xia et al., 2019). Many authors have shown that lysozyme is able to improve immunoglobulin A secretion and activate macrophage with a rapid riddance of bacterial diseases (Wang et al., 2005; Clarke et al., 2010; Dang et al., 2022). Thus, lysozyme is a natural antimicrobial protein considered a necessary component of innate immunity (Liu et al., 2010; Bozakova et al., 2020; Bastamy et al., 2024). According to these data, antibiotics can be replaced by lysozymes in the diet of poultry. Dietary supplementation with lysozyme has been reported to enhance immunity and non-specific immunity, maintain the gut's barrier function, and improve chickens' growth performance (Abdel-Latif et al., 2017). Also, lysozyme decreased the pathogen count in the ceca of chickens (Gong et al., 2017; Ma et al., 2017) and improved their gut antioxidant activity (Abdel-Latif et al., 2017). In swine, antibiotics as growth promoter at a sub-therapeutic level can be replaced by dietary lysozyme (Oliver and Wells, 2015). Adding of lysozyme in broiler chicken diets reduced the ileal count of *Clostridium perfringens* and their intestinal-induced lesions following the oral infection of these bacteria (Liu et al., 2010). Antibiotic growth promoters can be replaced by lysozyme to improve poultry production (Ma et al., 2017).

Mammalian milk, saliva, tears, and avian egg white contain lysozyme (Cao et al., 2015). The use of chicken egg lysozyme as a dietary supplementation is a vital tool to increase weight gain and the protection against diseases due to the functions of lysosome in the stimulation of immunity and potential against bacteria by altering the host–pathogen relationship (Hafez and Attia, 2020). In this way, dietary supplementation with chicken egg lysozyme improved the growth efficacy in rabbits (El-Deep et al., 2020) and weaned pigs (Oliver and Wells, 2013). The basal diet supplemented with chicken egg lysozyme enhanced blood metabolites, immunity, and total bacterial counts, while decreased the potential pathogen shedding and *E. coli* count in animals (Brundige et al., 2008; Oliver and Wells, 2013; El-Deep et al., 2020). Reports on the effect of the different types of exogenous lysozyme on chicken performance compared to growth promoters in the diet are rare. Therefore, the present study aimed to evaluate the impact of adding different types of lysozymes versus antibiotic growth promoters in the diet on the growth performance, hematological and blood biochemical parameters, immunity, antioxidant capacity, and intestinal microbial count in broiler chickens.

## 2 Materials and methods

The current study was performed in collaboration with the Animal, Poultry and Fish Production Department of the Faculty of Agriculture at Damietta University and a commercial chicken farm in Mansoura City, Dakahlia Governorate, Egypt. The “Directive 2010/63/EU of the European Parliament and of the council, of 22 September 2010, on the protection of animals used for scientific reasons” was followed in all procedures and experimental protocols.

### 2.1 Birds and experimental design

This experiment was conducted with three-hundred 1 d old, unsexed broiler chicks (Cobb-avian500). The experimental birds were allocated into five similar groups based on initial body weight (47.24 ± 1.27 g). Each group consisted of five replicates with 60 broilers per replicate. The first group (control) was fed a commercial control diet free of supplements. The control diet was supplemented with 0.2 g lincomycin, 1 g lysozyme, 25 mg egg lysozyme, and 50 mg egg lysozyme per kg of diet for the second, third, fourth, and fifth groups. Egg lysozyme was extracted from non-fertile egg white of chicken after the method by Ibrahim et al. (2011). Management of birds was suggested according to the guidelines of the *Cobb Broiler Management Guide*.

### 2.2 Feeding system

All birds were fed a commercial starter (Day 0–20) and finisher diet (Day 21–35) for broiler chickens (NRC, 1994). All diets were provided in a mash form throughout the trial. The chemical analyses of the starter and finisher diets (Table 1) were performed according to AOACAssociation of Official Agricultural Chemists methods (AOAC, 2016). Ad libitum feeding and fresh tap water were available for the experimental birds. The temperature was adjusted to 33 °C for the first 7 d of the experiment, and then the temperature was decreased by 3 °C per week until 21 °C. Artificial light was 24 h at 45 lx (lux) for the first 4 d of age. From days 5 to 35, the lighting schedule was a cycle of 18 h light and 6 h dark.

**Table 1:** Composition and analysis of the diet.

Ingredients	Starter	Finisher
Yellow corn	60.50	58.50
Soybean meal 48 %	30.80	35.30
Corn gluten meal 60 %	4.00	0.00
Soybean oil	0	2.20
Ground limestone	1.40	1.80
Dicalcium phosphate	2.35	1.25
Salt	0.35	0.35
Premix ${}^{*}$	0.30	0.30
DL-methionine	0.10	0.10
L-lysine	0.10	0.10
Coccidiostats	0.10	0.10
Total	100	100
Chemical analysis ${}^{**}$		
Crude protein, %	22.40	21.00
Metabolizable energy (kcal kg ${}^{-1}$ )	2950	3100
Calcium, %	1.05	1.01
Available phosphorus, %	0.45	0.45
Lysine, %	1.18	1.00
Methionine, %	0.49	0.42
Meth.+Cys., %	0.86	0.80

${}^{*}$ Premix at 0.30 % of the diet supplies, with the following per kilogram of the diets: vitamin A 1000 IU (international unit), vit. D ${}_{3}$ 2000 IU, vit. E 10 mg, vit. K 1 mg, vit. B ${}_{1}$ 5 mg, vit. B ${}_{2}$ 5 mg, vit. B ${}_{6}$ 1.5 mg, vit. B ${}_{12}$ 0.01 mg, folic acid 0.35 mg, biotin 0.05 mg, pantothenic acid 10 mg, niacin 30 mg, choline 250 mg, Fe 30 mg, Zn 50 mg, Cu 4 mg, and Se 0.1 mg. Meth.+Cys. represents methionine+cysteine. ${}^{**}$ According to NRC (1994).

### 2.3 Performance measurements 

Body weight (BW) and feed intake (FI) were determined at the beginning (1 d of age) and at the end of the experiment (35 d of age); then final body-weight gain (BWG), average FI, and feed conversion ratio (FCR; gram of feed per gram of gain) were evaluated. The number of dead birds was recorded during the experimental period, and the viability rate was calculated for each group.

### 2.4 Blood samples 

At the end of the experiment at 35 d of age, samples of blood (10 mL) were drawn from five birds per group at slaughter. The blood sample (5 mL) was kept in a sterile test tube containing anticoagulant (EDTA) for the quantification of hematological indices such as hemoglobin (Hb), counts of red and white blood cells (RBCs and WBCs), packed cell volume (PCV), lymphocytes ( L ), heterophils ( H ), and H/L ratio. Other blood samples (5 mL) without anticoagulant were left to clot at lab temperature and then centrifuged (3000 rpm, 15 min) to obtain blood serum and stored at - 20 °C into aliquots for individual biochemical estimations. Concentrations of total proteins, albumin, total cholesterol, triglycerides, high-density lipoproteins (HDL), glucose, urea, creatinine, calcium (Ca), phosphorus (P), and activities of aspartate and alanine aminotransferases (AST and ALT) were determined in blood serum. Serum total antioxidant capacity (TAC), glutathione (GSH), glutathione peroxidase (GPx), superoxide dismutase (SOD), and malondialdehyde (MDA) were also determined. Blood biochemistry and antioxidants were assessed calorimetrically using bio-diagnostic kits (Biodiagnostic, Cairo, Egypt), following the manufacturer's directions. Cytokines markers, including interferon-gamma (IFN γ ), interleukin 2 (IL-2), and interleukin 10 (IL-10), were measured in blood serum as described by ELnaggar et al. (2016).

### 2.5 Bacterial count

At slaughter time, samples of the median region of the intestine were taken from five birds per group for determination of the total bacterial count (ICMSF, 2011), *Salmonella*, *Escherichia coli*, and *Proteus* counts, according to ISO-6579: 2002 for food microbiology procedures (ISO standards catalogue 07.100.30; World Health Organization, 2010).

### 2.6 Carcass traits

The liver, gizzard, and heart were dissected and weighed for the five slaughtered birds from each group. The net carcass weight and edible offal weight, including liver without a gall bladder, heart, and skinned empty gizzard were recorded. The dressing rate (DR) was computed with the following equation: DR = (Weight of eviscerated carcass + weight of edible offal) / (preslaughter weight) × 100. Also, the weight of the breast and thighs was recorded and estimated as a percentage of the carcass weight.

### 2.7 Statistical analysis

The obtained data were statistically analyzed in a completely randomized design (one-way ANOVA) using SAS software (SAS, 2012). The following statistical model was used for parameters:

$$Y_{ij}=\mu +G_{i}+e_{ij},$$

where $Y_{ij}$ represents observed values, μ represents the general mean, $G_{i}$ represents the group impact, and $e_{ij}$ represents the residual error. Percentage values of dressing rate and breast and thigh percentages were analyzed by a $\chi^{2}$ test. Duncan's new multiple range test was used to separate the significant group differences at a level of 5 % ( p<0.05 ).

## 3 Results

### 3.1 Growth performance

The dietary impacts of lincomycin, commercial lysozyme, and chicken egg lysozyme levels on the growth performance of broiler chickens are presented in Table 2. Diets with all supplements had greater ( p<0.05 ) impacts on final body weight and weight gain at the end of the experiment compared to the control group. And supplementation of the diet with egg lysozyme (50 mg kg ${}^{-1}$ ) significantly increased ( p<0.05 ) feed intake. Lincomycin, commercial lysozyme and egg lysozyme (25 mg) significantly improved ( p<0.05 ) the FCR compared with control and egg lysozyme (50 mg) groups. The viability rate difference was insignificant ( p<0.05 , Table 2).

**Table 2:** Effect of lincomycin, commercial lysozyme, and chicken egg lysozyme levels on the growth performance parameters and viability rate of broiler chickens.

Item	Control	Treatments per kg diet				p value
		Lincomycin	Commercial lysozyme	Egg lysozyme	Egg lysozyme	
		(0.2 g)	(1 g)	(25 mg)	(50 mg)	
Initial body weight (g)	46.8 ± 1.59	46.7 ± 1.17	46.9 ± 1.18	46.5 ± 1.45	46.1 ± 0.95	0.9921
Final body weight, (g)	2060 ± 16.59 ${}^{c}$	2125 ± 16.50 ${}^{b}$	2163 ± 5.91 ${}^{\mathrm{ab}}$	2166 ± 5.99 ${}^{\mathrm{ab}}$	2202 ± 20.83 ${}^{a}$	<0.0001
Body weight gain (1–35 g per chick)	2013 ± 17.13 ${}^{c}$	2078 ± 16.12 ${}^{b}$	2116 ± 6.05 ${}^{\mathrm{ab}}$	2119 ± 15.69 ${}^{\mathrm{ab}}$	2156 ± 21.25 ${}^{a}$	0.1436
Feed intake (1–35 g per chick)	3039 ± 10.09 ${}^{b}$	3076 ± 11.58 ${}^{b}$	3066 ± 16.68 ${}^{b}$	3074 ± 17.29 ${}^{b}$	3218 ± 12.88 ${}^{a}$	<0.0001
Total FCR (g feed per g BWG)	1.51 ± 0.03 ${}^{a}$	1.48 ± 0.03 ${}^{b}$	1.45 ± 0.01 ${}^{c}$	1.45 ± 0.02 ${}^{c}$	1.49 ± 0.02 ${}^{\mathrm{ab}}$	0.0034
Viability rate	98.21	97.22	96.82	97.21	98.1	–

${}^{a,b,c}$ Means within the same row with different superscripts are significantly different ( p<0.05 ).

### 3.2 Hematological parameters

The dietary impacts of lincomycin, commercial lysozyme, and chicken egg lysozyme levels on hematological parameters are shown in Table 3. Experimental birds fed the dietary supplementation of commercial lysozyme and both levels of egg lysozyme showed a significant impact on Hb concentration and RBC count compared to the control or lincomycin groups ( p<0.05 ). The addition of lincomycin, commercial lysozyme, and both levels of egg lysozyme to the control diet significantly ( p<0.05 ) decreased the count of WBCs, heterophils, and the heterophils / lymphocyte ratio and markedly improved ( p<0.05 ) lymphocyte percent compared to the control diet. The PCV value was not affected significantly by lysozyme supplementation. Dietary supplementation with egg lysozyme (50 mg kg ${}^{-1}$ ) gives the best hematological parameters (Table 3).

**Table 3:** Effect of lincomycin, commercial lysozyme, and chicken egg lysozyme levels on hematological parameters of broiler chickens.

Item	Control	Treatments per kg diet				p value
		Lincomycin	Commercial lysozyme	Egg lysozyme	Egg lysozyme	
		(0.2 g)	(1 g)	(25 mg)	(50 mg)	
Hb (g dL ${}^{-1}$ )	12.7 ± 0.42 ${}^{b}$	13.5 ± 0.37 ${}^{b}$	13.9 ± 0.23 ${}^{a}$	14.1 ± 0.44 ${}^{a}$	14.1 ± 0.23 ${}^{a}$	0.0563
RBCs (10 ${}^{12}$ L ${}^{-1}$ )	1.94 ± 0.03 ${}^{c}$	2.01 ± 0.03 ${}^{\mathrm{bc}}$	2.04 ± 0.02 ${}^{b}$	2.10 ± 0.03 ${}^{a}$	2.11 ± 0.02 ${}^{a}$	0.0009
WBCs (10 ${}^{6}$ L ${}^{-1}$ )	27.4 ± 0.95 ${}^{a}$	22.1 ± 1.66 ${}^{b}$	23.1 ± 1.28 ${}^{b}$	21.1 ± 1.24 ${}^{b}$	20.7 ± 1.57 ${}^{b}$	0.0111
PCV (%)	30.4 ± 1.26	31.5 ± 1.19	30.8 ± 1.22	32.1 ± 0.82	32.1 ± 1.25	0.8191
Heterophils ( H , %)	25.8 ± 1.47 ${}^{a}$	19.4 ± 1.06 ${}^{\mathrm{bc}}$	21.1 ± 1.100 ${}^{b}$	17.3 ± 1.17 ${}^{c}$	16.7 ± 1.23 ${}^{c}$	<0.0001
Lymphocytes ( L , %)	52.8 ± 1.01 ${}^{b}$	59.4 ± 1.25 ${}^{a}$	60.1 ± 1.10 ${}^{a}$	62.1 ± 1.06 ${}^{a}$	62.6 ± 0.99 ${}^{a}$	<0.0001
H/L ratio	0.49 ± 0.03 ${}^{a}$	0.32 ± 0.02 ${}^{\mathrm{bc}}$	0.35 ± 0.02 ${}^{b}$	0.28 ± 0.02 ${}^{c}$	0.27 ± 0.02 ${}^{c}$	<0.0001

${}^{a,b,c}$ Means within the same row with different superscripts are significantly different ( p<0.05 ). Abbreviations: Hb, hemoglobin; RBCs, red blood cells; WBCs, white blood cells; PCV, Packed Cell Volume.

### 3.3 Biochemical parameters

The inclusion of lincomycin, commercial lysozyme, and chicken egg lysozyme levels on biochemical parameters in serum is shown in Table 4. Total protein, albumin, globulin, and glucose levels were increased in all supplemented groups compared to the control. The birds fed the diet containing egg lysozyme only at a 50 mg kg ${}^{-1}$ rate had significantly lower alpha-globulin, ALT, triglycerides, cholesterol, and urea levels and higher HDL concentration than the other groups. The effect of all supplements on beta- and gamma-globulin, AST, Ca, P, and creatinine was not altered ( p>0.05 , Table 4).

**Table 4:** Effect of lincomycin, commercial lysozyme, and chicken egg lysozyme levels on biochemical parameters of broiler chickens.

Item	Control	Treatments per kg diet				p value
		Lincomycin	Commercial lysozyme	Egg lysozyme	Egg lysozyme	
		(0.2 g)	(1 g)	(25 mg)	(50 mg)	
TP (g dL ${}^{-1}$ )	5.24 ± 0.09 ${}^{c}$	5.74 ± 0.07 ${}^{b}$	6.14 ± 0.05 ${}^{a}$	6.09 ± 0.04 ${}^{a}$	6.01 ± 0.03 ${}^{a}$	<0.0001
Al (g dL ${}^{-1}$ )	3.29 ± 0.03 ${}^{c}$	3.46 ± 0.04 ${}^{b}$	3.60 ± 0.02 ${}^{a}$	3.51 ± 0.05 ${}^{\mathrm{ab}}$	3.46 ± 0.02 ${}^{b}$	<0.0001
Gl (g dL ${}^{-1}$ )	1.95 ± 0.08 ${}^{c}$	2.28 ± 0.09 ${}^{b}$	2.53 ± 0.05 ${}^{a}$	2.59 ± 0.07 ${}^{a}$	2.55 ± 0.02 ${}^{a}$	<0.0001
Glucose (mg dL ${}^{-1}$ )	154 ± 1.57 ${}^{b}$	163 ± 1.35 ${}^{a}$	167 ± 1.80 ${}^{a}$	166 ± 1.61 ${}^{a}$	166 ± 1.65 ${}^{a}$	<0.0001
Calcium (mg dL ${}^{-1}$ )	8.57 ± 0.64	9.14 ± 0.80	9.28 ± 0.61	9.57 ± 0.65	9.14 ± 0.51	0.8660
Phosphorus (mg dL ${}^{-1}$ )	5.85 ± 0.86	5.16 ± 0.51	5.05 ± 0.47	4.92 ± 0.50	5.14 ± 0.59	0.8311
Trigly. (mg dL ${}^{-1}$ )	32.2 ± 1.27 ${}^{a}$	31.7 ± 0.83 ${}^{a}$	31.2 ± 0.97 ${}^{a}$	29.8 ± 1.37 ${}^{a}$	26.1 ± 1.14 ${}^{b}$	0.0044
Choles. (mg dL ${}^{-1}$ )	122 ± 1.58 ${}^{a}$	117 ± 1.60 ${}^{\mathrm{ab}}$	117 ± 1.97 ${}^{\mathrm{ab}}$	117 ± 1.48 ${}^{\mathrm{ab}}$	116 ± 2.27 ${}^{b}$	0.0546
HDL (mg dL ${}^{-1}$ )	96.2 ± 2.60 ${}^{b}$	98.5 ± 2.59 ${}^{\mathrm{ab}}$	96.7 ± 1.85 ${}^{\mathrm{ab}}$	98.2 ± 1.82 ${}^{\mathrm{ab}}$	103 ± 1.96 ${}^{a}$	0.0525
Urea (mg dL ${}^{-1}$ )	10.28 ± 0.74 ${}^{a}$	8.85 ± 0.91 ${}^{\mathrm{ab}}$	8.28 ± 0.97 ${}^{\mathrm{ab}}$	8.43 ± 0.65 ${}^{\mathrm{ab}}$	6.78 ± 0.69 ${}^{b}$	0.0541
Creat. (mg dL ${}^{-1}$ )	0.96 ± 0.02	0.92 ± 0.04	0.88 ± 0.02	0.91 ± 0.03	0.92 ± 0.02	0.4030
AST (IU dL ${}^{-1}$ )	68.8 ± 1.03	67.4 ± 1.06	64.8 ± 1.16	65.7 ± 1.58	67.1 ± 1.50	0.2579
ALT (IU dL ${}^{-1}$ )	52.8 ± 1.38 ${}^{a}$	50.5 ± 1.19 ${}^{\mathrm{ab}}$	48.7 ± 1.06 ${}^{b}$	49.8 ± 1.61 ${}^{\mathrm{ab}}$	47.3 ± 1.19 ${}^{b}$	0.0582

${}^{a,b,c}$ Means within the same row with different superscripts are significantly different ( p<0.05 ). Abbreviations: TP, total proteins; Al, albumin; Gl, globulin; Trigly., triglycerides; Choles., cholesterol; HDL, high-density lipoprotein; AST, aspartate aminotransferase; ALT, alanine aminotransferase; Creat., creatinine.

### 3.4 Antioxidants capacity and immunity

Data on antioxidant capacity markers and immunity constituents in blood serum are shown in Table 5. Supplementation of lincomycin, commercial lysozyme, and chicken egg lysozyme levels significantly increased TAC, GSH, GPx and SOD and significantly decreased MDA levels compared to the control diet. The immune response, in terms of IFN and IL-2, was significantly improved by lincomycin, commercial lysozyme, and chicken egg lysozyme levels addition as compared to the control diet, but IL-10 was significantly increased only by egg lysozyme supplementation at a level of 50 mg kg ${}^{-1}$ diet relative to other supplementations and the control diet. Addition of egg lysozyme (50 mg) showed a superiority in antioxidant activity and immunity of broiler chickens (Table 5).

**Table 5:** Effect of lincomycin, commercial lysozyme, and chicken egg lysozyme levels on antioxidants capacity and immunity of broiler chickens.

Item	Control	Treatments per kg diet				p value
		Lincomycin	Commercial lysozyme	Egg lysozyme	Egg lysozyme	
		(0.2 g)	(1 g)	(25 mg)	(50 mg)	
Antioxidants capacity						
TAC (ng mL ${}^{-1}$ )	0.28 ± 0.02 ${}^{c}$	0.31 ± 0.17 ${}^{b}$	0.33 ± 0.01 ${}^{b}$	0.34 ± 0.01 ${}^{b}$	0.39 ± 0.01 ${}^{a}$	<0.0001
GSH (IU L ${}^{-1}$ )	0.16 ± 0.01 ${}^{c}$	0.20 ± 0.01 ${}^{b}$	0.21 ± 0.02 ${}^{b}$	0.22 ± 0.02 ${}^{b}$	0.28 ± 0.01 ${}^{a}$	<0.0001
GPx (IU L ${}^{-1}$ )	27.8 ± 1.33 ${}^{c}$	41.2 ± 0.86 ${}^{b}$	41.5 ± 1.46 ${}^{b}$	40.5 ± 0.87 ${}^{b}$	49.5 ± 0.95 ${}^{a}$	<0.0001
SOD (IU L ${}^{-1}$ )	0.22 ± 0.01 ${}^{d}$	0.27 ± 0.02 ${}^{c}$	0.30 ± 0.01 ${}^{\mathrm{bc}}$	0.31 ± 0.03 ${}^{\mathrm{ab}}$	0.33 ± 0.01 ${}^{a}$	<0.0001
MDA (nmol mL ${}^{-1}$ )	0.40 ± 0.01 ${}^{a}$	0.31 ± 0.01 ${}^{b}$	0.31 ± 0.02 ${}^{b}$	0.29 ± 0.01b ${}^{c}$	0.26 ± 0.02 ${}^{c}$	<0.0001
Immunity						
IFN γ (pg mL ${}^{-1}$ )	3.76 ± 0.09 ${}^{c}$	4.06 ± 0.03 ${}^{\mathrm{ab}}$	4.03 ± 0.03 ${}^{b}$	4.16 ± 0.03 ${}^{\mathrm{ab}}$	4.27 ± 0.10 ${}^{a}$	0.0002
IL-2 (pg mL ${}^{-1}$ )	6.33 ± 0.03 ${}^{d}$	6.75 ± 0.06 ${}^{c}$	6.98 ± 0.04 ${}^{b}$	7.06 ± 0.04 ${}^{b}$	7.21 ± 0.03 ${}^{a}$	<0.0001
IL-10 (pg mL ${}^{-1}$ )	16.1 ± 1.32 ${}^{b}$	17.4 ± 0.75 ${}^{\mathrm{ab}}$	17.3 ± 0.68 ${}^{\mathrm{ab}}$	18.4 ± 1.13 ${}^{\mathrm{ab}}$	19.6 ± 0.65 ${}^{a}$	0.0116

${}^{a,b,c}$ Means within the same row with different superscripts are significantly different ( p<0.05 ). Abbreviations: TAC, total antioxidants capacity; GSH, glutathione; GPx, glutathione peroxidase; SOD, superoxide dismutase; MDA, malondialdehyde; IFN γ , interferon-gamma; IL-2, interleukin 2; IL-10, interleukin 10.

### 3.5 Intestinal bacterial counts

The intestinal bacterial counts in terms of total bacteria count, *Salmonella*, *E. coli*, and *Proteus* of broiler chickens fed diets with lincomycin; commercial lysozyme, and chicken egg lysozyme are presented in Table 6. The total bacterial count was significantly increased, and *Salmonella*, *E. coli*, and *Proteus* counts were significantly decreased in chickens by all dietary supplementations compared to the control diet.

**Table 6:** Effect of lincomycin, commercial lysozyme, and chicken egg lysozyme levels on intestinal bacterial counts of broiler chickens.

Item	Control	Treatments per kg diet				p value
		Lincomycin	Commercial lysozyme	Egg lysozyme	Egg lysozyme	
		(0.2 g)	(1 g)	(25 mg)	(50 mg)	
Total bacterial count	3.88 ± 0.03 ${}^{a}$	3.54 ± 0.04 ${}^{b}$	3.37 ± 0.04 ${}^{c}$	3.31 ± 0.03 ${}^{c}$	3.17 ± 0.03 ${}^{d}$	<0.0001
*Salmonella*	1.28 ± 0.02 ${}^{a}$	1.03 ± 0.01 ${}^{b}$	1.04 ± 0.08 ${}^{b}$	1.04 ± 0.04 ${}^{b}$	0.91 ± 0.02 ${}^{b}$	0.0093
*E. coli*	1.44 ± 0.02 ${}^{a}$	1.10 ± 0.03 ${}^{b}$	0.83 ± 0.02 ${}^{d}$	0.88 ± 0.02 ${}^{\mathrm{cd}}$	0.95 ± 0.02 ${}^{c}$	<0.0001
*Proteus*	0.95 ± 0.03 ${}^{a}$	0.87 ± 0.01 ${}^{b}$	0.85 ± 0.01 ${}^{b}$	0.86 ± 0.02 ${}^{b}$	0.77 ± 0.02 ${}^{c}$	<0.0001

${}^{a,b,c,d}$ Means within the same row with different superscripts are significantly different ( p<0.05 ).

### 3.6 Carcass traits

Results of dressing rate and some carcass trait percentages in the experimental groups are presented in Table 7. Results revealed significant improvements in dressing rate and breast weight percentage only by adding egg lysozyme in the broiler chicken diet at 50 mg kg ${}^{-1}$ compared to other additions and supplement-free diets. Thigh weight percentage was increased only by commercial lysozyme supplementation compared to other supplementations and control groups (Table 7).

**Table 7:** Effect of lincomycin, commercial lysozyme, and chicken egg lysozyme levels on some carcass traits of broiler chickens.

Item	Control	Treatments per kg diet				p value
		Lincomycin	Commercial lysozyme	Egg lysozyme	Egg lysozyme	
		(0.2 g)	(1 g)	(25 mg)	(50 mg)	
Dressing rate	73.3 ± 2.34 ${}^{b}$	72.9 ± 2.64 ${}^{b}$	73.6 ± 2.45 ${}^{\mathrm{ab}}$	74.1 ± 3.10 ${}^{\mathrm{ab}}$	76.8 ± 1.78 ${}^{a}$	<0.0001
Breast weight (%)	42.1 ± 3.29 ${}^{a}$	40.6 ± 4.99 ${}^{\mathrm{ab}}$	34.7 ± 6.19 ${}^{b}$	40.1 ± 6.35 ${}^{\mathrm{ab}}$	42.2 ± 6.89 ${}^{a}$	<0.0001
Thighs weight (%)	31.2 ± 1.47 ${}^{b}$	32.3 ± 6.96 ${}^{b}$	38.9 ± 6.04 ${}^{a}$	33.9 ± 5.99 ${}^{b}$	34.6 ± 5.29 ${}^{b}$	<0.0001

${}^{a,b}$ Means within the same row with different superscripts are significantly different ( p<0.05 ).

## 4 Discussion

In Egypt, chicken meat is an important source of animal protein for consumers. As growth promoters, dietary antibiotics are frequently used for poultry to enhance weight gain and feed utilization and to reduce bacterial infections (Salaheen et al., 2017; Selaledi et al., 2020). Despite this, their usage is banned in some countries of the world, and there are attempts to prevent or reduce this usage worldwide. The presence of safe and effective alternatives to conventional antibiotics may have a competitive advantage for poultry producers in relation to antibiotic use (El Basuini et al., 2023). Lysozyme is one of the most promising antibiotic alternatives. It is widely distributed in tissues and secretions of animals and plays an important role fighting against several pathogenic bacteria, which gives a positive output in the Gram stain test via hydrolyzing their cell wall's β -1,4-glycosidic linkage between N-acetyl muramic acid and N-acetyl glucosamine (Ibrahim et al., 1994; Abdel-Latif et al., 2024). In this way, using chicken egg lysozyme as a dietary supplementation benefits weight gain and prevents poultry diseases (Asante et al., 2019; Zou et al., 2019). Applying growth agents alters intensive poultry production via their advantages of improving gut health, decreasing subclinical infections, and promoting growth performance parameters (Rafiq et al., 2022).

The obtained results in our study revealed that broiler chickens fed diets containing lincomycin, commercial lysozyme, and both levels of chicken egg lysozymes had improved final BW and BWG, but FCR of birds fed a diet containing 50 mg of egg lysozyme per kg of diet did not differ from that of the supplement-free diet (control), due to increasing their feed intakes as compared to other groups. Other effective natural dietary supplementations to replace antibiotics (pro-,pre-,phyto-, and sym-biotic, spirulina, or their combinations) were reported to improve growth efficiency and the viability rate of broilers (Alam and Ferdaushi, 2018; Ferdous et al., 2019). Our results agree with improving the live body weight and weight gain of broilers fed a diet supplemented with exogenous lysozyme (Abdel-Latif et al., 2017) as well as FCR and weight gain (Liu et al., 2010) of broiler chickens. Dietary supplementation with chicken egg lysozyme enhanced ( p<0.05 ) growth performance and FCR in growing rabbits (El-Deep et al., 2020) and weaned pigs (Oliver and Wells, 2013). Adding lysozyme at 50 and 100 mg kg ${}^{-1}$ to the diet of broiler chickens improves growth performance (Hassan et al., 2023). Body weight, feed conversion ratio, and body weight gain were markedly ( p<0.05 ) improved in both avilamycin and lysozyme groups in relation to a control group (Abdel-Latif et al., 2024). The growth-promoting action of chicken egg lysozyme in our study was attributed to improving gut antioxidant and antimicrobial properties and increasing the nutrient digestibility in the gut of animals. Such benefits can improve the absorption of nutrients in the intestine (Georgieva et al., 2000; Hussein et al., 2020).

We detected a change in birds' hematological and biochemical parameters in all groups to provide some markers of the health status affected by different feed additives (Musco et al., 2019; El-Deep et al., 2020). We found that dietary commercial lysozyme and chicken egg lysozymes significantly improved all the studied hematological parameters, the best being egg lysozyme (50 mg kg ${}^{-1}$ diet). Animal feed additives must have high feeding values, lead to good health status, and be without harmful effects to save animal products for human consumption (Phillips et al., 2003). Enhancement in the RBC count was associated with improving the health and physiological statuses of birds fed chicken egg lysozymes (Musco et al., 2019). However, increasing the WBC count and lymphocytes percentage may be mainly due to the action of egg lysozymes from chicken to improve the immune response, so chicken egg lysozymes are a good source of antioxidants (El-Deep et al., 2020). Chicken lysosomes can prevent the harmful effects of free radicals and may decrease toxicity and improve liver health (Robertson et al., 2016).

The results indicated that all supplements in broiler rations could improve blood biochemical indices such as total protein and their fractions, leading to better protein metabolism with a pronounced effect of 50 mg lysozyme on increasing alpha-globulin. The increasing concentration of serum total protein in egg lysozyme groups may be attributed to the fact that chicken egg white contains high-quality proteins and bioactive peptides (Huang et al., 2010; Chen and Zhang, 2012). Our supplementation of lysozymes increased the concentration of globulin compared to the control group. Similar results were reported in the exogenous dietary lysozyme by Abdel-Latif et al. (2017) in broiler chickens. It is of interest to found no adverse impacts on the functions of the liver and kidney of chickens fed all supplements, except for egg lysozyme (50 mg kg ${}^{-1}$ ) supplementation, which exhibited more improvement in liver and kidney functions by reducing ALT activity and urea concentration in blood serum, indicating the safety of egg lysozyme supplementation. Based on these results, egg lysozyme is an excellent source of antioxidants that can protect body cells from free radicals, reduce toxicity, and possibly even safeguard liver health by preventing liver damage (Robertson et al., 2016). In this context, Oliver et al. (2014) found that chicken egg lysozyme consumption reduced the circulating urea and improved protein accretion.

Lipid profiles in the serum were improved only in birds fed a diet with chicken egg lysozyme (50 mg kg ${}^{-1}$ ) in terms of considerable reduction in triglycerides and cholesterol levels and increasing HDL level. The results of our findings agree with Amer et al. (2023), who reported that increased levels of lysozyme reduced the total cholesterol, triglycerides, and low-density lipoprotein (LDL), while considerably raising HDL indicating a hypolipidemic effect of lysozyme. In rabbits, dietary lysozyme had a positive impact on reducing lipid profile in serum compared with the control group (Abu Hafsa et al., 2022). Blood HDL cholesterol removes more dangerous cholesterol types from blood circulation. The results support the lysozyme role as a health-promoting supplement in rabbit diet (Martini and Pallottini, 2007).

In the present study, all dietary supplementations significantly increased antioxidants capacity, the level of TAC, and activity of GSH, GPx, and SOD, while significantly decreased the level of MDA compared with the control diet. Similarly, significant improvements were observed in antioxidant enzymes and MDA in the lysozyme (50 and 100 mg kg ${}^{-1}$ ) groups (Hassan et al., 2023).

The increase in TAC is a biomarker for increasing the scavenging oxidative activity against free radicals (Kambayashi et al., 2009). Supplementation of lysozyme was found to increase the SOD activity in rabbits (Abu Hafsa et al., 2022). Also, overexpression of SOD and GPx activities in the intestine was increased by exogenous lysozyme as a marker of the intestinal detoxification status increment against various xenobiotics (Abdel-Latif et al., 2017). Dietary supplementation of lysozyme exhibited an increase in the blood SOD activity and decrease in the level of MDA along with an increasing number of goblet cells and length of microvilli in the median region of the intestine (Chen and Zhang, 2012). Chicken egg white proteins may increase the release and the biological activity of bioactive peptides and the activity of antioxidants (Huang et al., 2010; Chen and Zhang, 2012).

Lysozyme is an important non-specific immune-modulating factor (Ferraboschi and Ciceri, 2021). The inclusion of chicken egg lysozyme to the diet as a feed additive is considered a stimulant of immunity and potential against bacteria due to its role in the host–pathogen relationship changes (Hafez and Attia, 2020; Dawood et al., 2024). Obminìska-Mrukowicz (2022) studied the immunomodulatory properties of lysozyme and indicated the pharmacological protection of immunohomeostasis during bacterial and viral infections. Furthermore, the last author stated that lysozyme could be applied to improve the immune response during vaccination and for the compensation of the impaired immune system due to immunosuppressive factors. In the present study, the best immunity parameters were obtained in birds fed a diet with egg lysozyme (50 mg kg ${}^{-1}$ ) regarding the highest level of IFN γ , IL-6, and IL-10 in the blood serum of birds. Similarly, supplementation of exogenous lysozyme (90 g t ${}^{-1}$ ) improved the non-specific immunity of broiler chickens (Abdel-Latif et al., 2017). In the same context, microbial lysozyme could improve intestinal integrity and immune responses in broiler chickens (Du and Guo, 2021; Bastamy et al., 2024). Lysozyme administration in diets of broiler chickens (50 and 100 mg kg ${}^{-1}$ ) and layer chickens (200 and 300 mg kg ${}^{-1}$ ) improved intestinal morphology and immune efficiency when compared to the control group (Hassan et al., 2023; Sindaye et al., 2023).

Lysozyme supplementation showed some degree of improved immunity in rabbits (El-Deep et al., 2020). Augmentation of the immune response in adult pigeons with exogenous lysozyme (van de Crommenacker et al., 2010) and stimulation of the humoral defense mechanisms in rainbow trout with dimerized lysozyme (Siwicki, et al., 1998) were also reported. An enhancement in the immune response was reported by supplementation of chicken egg lysozymes in pigs' diets (Oliver et al., 2014). Chicken egg lysozyme supplementation improved chickens' immune systems due to increasing antioxidant activity (Fritz et al., 2009). Feeding chicken egg lysozyme modulates serum antibodies (IgA) production and the tunica mucosa in growing pigs' intestines (Zou et al., 2019).

The current study indicated that all dietary supplements improved the intestinal total microbial count, while decreasing the count of *Salmonella*, *E. coli*, and *Proteus* compared to the control diet. The results of our findings agree with Abdel-Latif et al. (2024), who found that supplementation of lysozyme increased the total lactobacillus count and decreased the total coliform count at 21st and 35th day of age, which might lead to improved microbial balance in the body. In the same context, feeding lysozyme to broiler chickens reduced the count of *E. coli* in the ileum compared with feeding virginiamycin to birds. Dietary lysozyme at 100 ppm can change intestinal microbiota of broiler chickens (Gong et al., 2017). Lysozyme administration in diets of broiler chickens at 50 and 100 mg kg ${}^{-1}$ reduced the pathological lesions caused by *E. coli* infections (Hassan et al., 2023). The intestinal bacterial counts are vital in increasing the digestive process and improving health status. El-Deep et al. (2020) reported a similar trend in growing rabbits, who found a decrease in intestinal *E. coli* count. The beneficial impact of egg lysozyme on the intestinal total count of bacteria was attributed to the properties of chicken lysosome as antimicrobial, antioxidant, anti-inflammatory, and immune-modulatory properties (Abdel-Latif et al., 2017), indicating the good health status of birds. Decreasing *Salmonella*, *E. coli*, and *Proteus* counts can be attributed to the property of lincomycin and different types of lysozymes to inhibit the growth of pathogenic bacteria, consequently improving the host health (May et al., 2012; Ma et al., 2017), especially the use of chicken egg lysozyme which reduced the *E. coli* count in pigs (Brundige et al., 2008). Furthermore, the antibacterial activity of chicken egg lysozyme is via hydrolysis of the peptidoglycan in the cell walls of pathogenic microbes (Ibrahim et al., 2011; Du and Guo, 2021).

According to the obtained results, birds fed all supplements showed improved growth efficiency; in particular, those fed egg lysozyme diets during the growing period (1–35 d) were associated with augmentation of hematological parameters, improving protein metabolism, the profile of lipids, and function of liver and kidney, antioxidant status, immunity, and intestinal bacterial counts. The enhancement in growth performance led to the highest carcass quality parameters in birds fed egg lysozyme at a 50 mg kg ${}^{-1}$ diet, showing the highest dressing rate and breast weight percentage.

## 5 Conclusions

Chicken egg lysozyme supplementation to broiler chickens' diet enhances the growth performance, antioxidant capacity, immunity, and intestinal health of chicken broilers. Therefore, chicken egg lysozyme (50 mg kg ${}^{-1}$ ), as a feed additive in broiler chicken diet, is promising and can replace antibiotics.

## Data Availability

The data presented in this study are available free of charge for any user upon reasonable request from the corresponding author.
